# Semen Modulates Inflammation and Angiogenesis in the Reproductive Tract of Female Rabbits

**DOI:** 10.3390/ani10122207

**Published:** 2020-11-25

**Authors:** Jaume Gardela, Amaia Jauregi-Miguel, Cristina A. Martinez, Heriberto Rodríguez-Martinez, Manel López-Béjar, Manuel Álvarez-Rodríguez

**Affiliations:** 1 Department of Biomedical and Clinical Sciences (BKV), Division of Children’s and Women Health (BKH), Obstetrics and Gynecology, Linköping University, 58185 Linköping, Sweden; jaume.gardela@uab.cat (J.G.); cristina.martinez-serrano@liu.se (C.A.M.); heriberto.rodriguez-martinez@liu.se (H.R.-M.); 2 Department of Animal Health and Anatomy, Veterinary Faculty, Universitat Autònoma de Barcelona, 08193 Bellaterra, Spain; manel.Lopez.Bejar@uab.cat; 3 Division of Molecular Medicine and Virology (MMV), Linköping University, 58185 Linköping, Sweden; amaya.jauregi.miguel@liu.se; 4 College of Veterinary Medicine, Western University of Health Sciences, Pomona, CA 91766, USA

**Keywords:** gene expression, endometrium, oviduct, spermatozoa, seminal plasma, inflammation, angiogenesis, rabbit

## Abstract

**Simple Summary:**

In mammals, the expression of regulatory genes is modified by the interaction between semen and the female reproductive tract. This study intends to unveil how mating or insemination with sperm-free seminal plasma, as well as the presence of preimplantation embryos, affects inflammation and angiogenesis in different segments of the reproductive tract of female rabbits. Gene expression of anti-inflammatory cytokines and angiogenesis mediators was analyzed in segmented tracts (cervix to infundibulum) in response to mating and sperm-free seminal plasma infusion. Moreover, the gene expression at different times post-mating was also analyzed. Results showed that gene expression changes were mainly localized in the uterus in the natural mating group, describing a clear temporal variation, while limited to the oviduct in the sperm-free seminal plasma group. These changes suggest an early response in the uterus and late modulation in the oviduct, distinctly demonstrating that semen and seminal plasma, through their interaction with the female reproductive tract, can differentially modulate the expression of anti-inflammatory and angiogenesis mediators.

**Abstract:**

The maternal environment modulates immune responses to facilitate embryo development and ensure pregnancy. Unraveling this modulation could improve the livestock breeding systems. Here it is hypothesized that the exposure of the female rabbit reproductive tract to semen, as well as to early embryos, modulates inflammation and angiogenesis among different tissue segments. qPCR analysis of the gene expression changes of the anti-inflammatory interleukin-10 (IL10) and transforming growth factor beta family (TGFβ1–3) and the angiogenesis mediator vascular endothelial growth factor (VEGF-A) were examined in response to mating or insemination with sperm-free seminal plasma (SP). Reproductive tract segment (cervix to infundibulum) samples were obtained in Experiment 1, 20 h after gonadotropin-releasing hormone (GnRH) stimulation (control), natural mating (NM) or vaginal infusion with sperm-free SP (SP-AI). Additionally, segmented samples were also obtained at 10, 24, 36, 68 or 72 h after GnRH-stimulation and natural mating (Experiment 2). The results of gene expression, analyzed by quantitative PCR, showed that NM effects were mainly localized in the uterine tissues, depicting clear temporal variation, while SP-AI effects were restricted to the oviduct. Changes in anti-inflammatory and angiogenesis mediators indicate an early response in the uterus and a late modulation in the oviduct either induced by semen or preimplantation embryos. This knowledge could be used in the implementation of physiological strategies in breeding systems to face the new challenges on rabbit productivity and sustainability.

## 1. Introduction

Considered either laboratory animals, pets, invasive species or livestock, the rabbit (*Oryctolagus cuniculus*) is one of the most versatile and multipurpose animal species bred by humans [1,2,3]. Subjected today to intense production for meat and fur, does are affected by requirements on fertility and lifespan [4]. Contrary to other livestock species, rabbits require the generation of genital-somatosensory signals during mating to generate the preovulatory peak of gonadotropin-releasing hormone (GnRH) [5,6]. The consequent release of luteinizing hormone from the anterior pituitary induces ovulation [5]. Despite requiring hormonal stimulation if artificial insemination (AI) is used [7], this technique is extensively employed, giving similar or better results than natural mating [8] when using fresh or cooled semen [9]. The success of AI with diluted semen in several species suggest that seminal plasma (SP) components, excluding spermatozoa, are not required for pregnancy [10]. However, in some species, such as rodents or pigs, reproductive success and pregnancy can be jeopardized when the SP signaling is disrupted [10]. Several studies demonstrated that SP has multiple effects on the female reproductive tract essential for conceptus and pregnancy, improving reproductive performance [10].

In different species of mammals, the direct interaction between the female reproductive tract and semen, as well as the sensorial stimulation during the act of mating [11], induce molecular and cellular modifications into the female reproductive tract [12,13,14], modulating the immune system and optimizing the reproductive outcomes [15]. The immune response generated as a result of mating leads to a suitable environment for embryo survival, implantation success, optimal fetal and placental development and the overall reproductive process [16].

The first description of female post-mating inflammation responses was made by observing rabbits in 1952 [17]. The observations of McDonald and collaborators showed a leucocytic influx in the uterus in response to SP but not by sterile spermatozoa administration [17]. Later studies demonstrated that both SP and spermatozoa triggered a uterine leucocytic response in rabbits [18,19]. Contrary to the vagina, cervix and uterus, the oviduct seems to respond to insemination without a reduction of the spermatozoa population at the time of ovulation [20], providing the optimal conditions for spermatozoa survival until fertilization takes place [7]. The interaction between spermatozoa and oviductal epithelial cells could regulate the immunological environment of the oviduct-inducing anti-inflammatory cytokines [21], like transforming growth factor beta 1 (TGFβ1) or interleukin-10 (IL10), protecting spermatozoa from immune responses [13].

The transforming growth factor beta (TGFβ) family regulates many cellular activities, including cell growth, proliferation, differentiation, tissue remodeling, extracellular matrix formation, control of cell surface molecules, immunoregulation, angiogenesis and apoptosis [22]. The TGFβ belong to a large superfamily of cytokines composed of three 25 kDa homodimeric proteins (TGFβ1–3) whose biological effects are mediated by three transforming growth factor beta receptors (TGFβR1–3) [22,23]. The IL10, first described as cytokine synthesis inhibitory factor or CSIF [24], is a cytokine that inhibits inflammatory responses preventing the secretion of inflammatory cytokines and regulating the differentiation and proliferation of immune cells [25].

The vascular endothelial growth factor (VEGF, also referred to as VEGF-A), a heparin-binding homodimeric glycoprotein, is a mitogen for endothelial cells and a potent inducer of angiogenesis [26] that also promotes vascular permeability [27]. VEGF protein family includes other factors like VEGF-B, VEGF-C, VEGF-D, VEGF-E, VEGF-F and placental growth factor (P1GF) [28,29]. VEGF-A is expressed in the endometrium of several species [30,31,32], being proposed in the rabbit as a local signal between the implantation embryo and vascular structures in the receptive endometrium [33].

This study aimed to determine whether natural mating and/or sperm-free SP infusion modulates the expression of anti-inflammatory cytokines and angiogenesis mediator genes (*IL10*, *TGFβ1–3* and *VEGF-A*) in the maternal environment of the doe at 20 h post-exposure. Moreover, we determine the expression of these mediators when early embryo development occurs along the reproductive tract of the doe up to 72 h post-mating. Additionally, we analyzed the spatial changes of these genes along the tubular reproductive tract of the doe.

## 2. Materials and Methods

### 2.1. Ethics Statement

Rabbits were handled according to the Spanish Royal Decree 1201/2005 (BOE, 2005: 252:34367-91) and the Directive 2010/63/EU of the European Parliament and of the Council of 22 September 2010 on the protection of animals used for scientific purposes (2010; 276:33–79). The Committee of Ethics and Animal Welfare of the Universitat Autónoma de Barcelona, Spain, approved this study (Expedient #517).

### 2.2. Animals and Experimental Design

Animals were housed in the nucleus colony at the farm of the Institut de Recerca i Tecnologia Agroalimentaries (IRTA-Torre Marimon, Caldes de Montbui, Barcelona, Spain) under a controlled photoperiod and temperature [34]. Single cage equipped with plastic footrests, a feeder (restricted to 180 g/day of an all-mash pellet) and nipple-drinkers (ad libitum access to water) were used for each rabbit.

Six New Zealand White (NZW) adult bucks (from seven to thirteen months old) were included in the study. At 4.5 months of age, the bucks were started to be trained with a homemade polyvinyl chloride artificial vagina at 50 °C to ensure 41–42 °C at the time of semen collection. One ejaculate was collected per male, discarding ejaculates that contained urine and calcium carbonate deposits on visual inspection.

**Figure 1:**
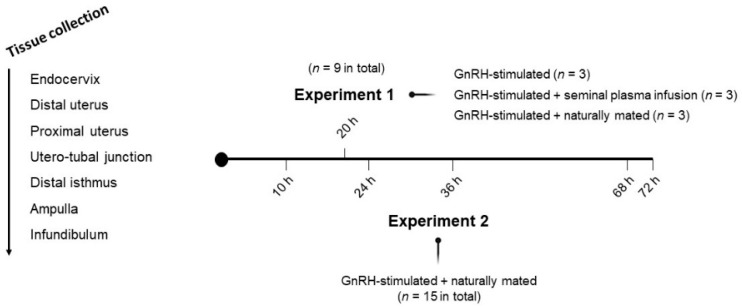
Representation of the experimental design and tissue sections obtained from does. Sequential tissue segments derived from does were endocervix, distal uterus, proximal uterus, utero-tubal junction, distal isthmus, ampulla and infundibulum. Intramuscular injection of 0.03 mg gonadotropin-releasing hormone (GnRH) was used to induce ovulation in all groups of both experiments.

Two separate experiments were performed in this study (Figure 1). In Experiment 1, 9 NZW adult does were randomly allocated into three experimental groups. Sequential segments of the right side of female reproductive tracts were retrieved after 20 h post-induction of the ovulation with 0.03 mg GnRH (Fertagyl^®^, Esteve Veterinaria, Barcelona, Spain) intramuscularly (im) (control of the ovulation; control, *n* = 3), 20 h post-induction of the ovulation with 0.03 mg GnRH im and sperm-free SP vaginal infusion (SP-AI, *n* = 3) and 20 h post-induction of the ovulation with 0.03 mg GnRH im and natural mating (NM, *n* = 3). In Experiment 2, 15 NZW adult does were sequentially euthanized at 10, 24, 36, 68 or 72 h post-induction of the ovulation with 0.03 mg GnRH im and natural mating (*n* = 3/collection time). The 10 h group was established as the reference group to compare the ovulatory moment (10 h post-mating) with some different time-point steps of the preimplantation embryo development: 2–4 cell embryo (24 h), 8-cell embryo (36 h), early morula (68 h) and morula (72 h).

### 2.3. Mating and Semen Collection

Two randomly selected bucks were used to mate the does included in the mating group of Experiment 1 and Experiment 2 after the hormonal induction of the ovulation with 0.03 mg GnRH im. Additionally, semen was collected from the same rabbit bucks through an artificial vagina, as described above. The sperm-free SP was obtained after centrifugation at 2000× *g* for 10 min and checked for the absence of spermatozoa. The harvested sperm-free SP was immediately used for sperm-free SP vaginal infusions of Experiment 1.

### 2.4. Tissue Sample Collection

For each experimental condition, seven consecutive tissue sections from the female reproductive tracts (endocervix, distal uterus, proximal uterus, utero-tubal junction, ampulla, isthmus and infundibulum; Figure 1) were obtained after the euthanasia of the does [34]. The tissue segments were retrieved and stored in RNAlater solution (Thermo Fisher Scientific, Waltham, MA, USA) at −80 °C.

Before tissue segmentation of does included in experiment 2, the oviduct was isolated and flushed (phosphate-buffer saline supplemented with 5% fetal calf serum and 1% antibiotic-antimycotic solution) to collect the embryos, which were examined by number and developmental stage. The number of ovulated ovarian follicles (5.08 ± 2.06 follicles, mean ± standard deviation (SD)) and embryos (4.67 ± 3.14, mean ± SD) were counted for each side of the reproductive organs, as previously published elsewhere [34]. Briefly, at 24 h (2 and 4-cell), 36 h (8-cell stage), 68 h (early morula) and 72 h (morula), the embryos were collected by the method described above.

### 2.5. Quantitative PCR Analyses

Briefly, total RNA was extracted from the tissue segments following a TRIzol-based protocol [34]. The RNA concentration of the extracts was determined from the absorbance of 260 nm with Thermo Scientific NanoDrop^TM^ 2000 (Fisher Scientific, Gothenburg, Sweden). The quality of the RNA was determined by the Agilent 2100 Bioanalyzer (Agilent Technologies, Palo Alto, CA, USA), using the samples with an RNA integrity number higher than 8. High-Capacity RNA-to-cDNA™ Kit (Applied Biosystems™, Foster City, CA, USA) was used to synthesize the first-strand cDNA for quantitative polymerase chain reaction (qPCR) analyses (CFX96™; Bio-Rad Laboratories, Inc; Hercules, CA, USA). Following our previous qPCR protocol [34], the gene relative expression levels were quantified using the Pfaffl method [35]. The primer sequences, product sizes and efficiencies are shown in Table 1. For the *β-ACTIN* gene, commercial gene-specific PCR primers for rabbit samples were used (PrimePCR™SYBR^®^ Green Assay: ACTB, Rabbit; Bio-Rad Laboratories, Inc., Hercules, CA, USA). Product sizes were confirmed by loading the amplicons in an agarose gel using a gel imaging system (ChemiDoc XRS+ System, BioRad Laboratories, Inc., Hercules, CA, USA).

### 2.6. Statistical Analyses

CFX Maestro™ 1.1 software version 4.1.2433.1219 (Bio-Rad Laboratories, Inc; Hercules, CA, USA) was used to export all data. Normal distribution and homoscedasticity of the data were analyzed using the Shapiro–Wilk normality test and Levene’s test. Non-normal data distribution was restored using Log(x) transformation prior to analysis. R version 3.6.1. [36] was used to conduct the statistical analyses with *nlme* [37] to perform linear mixed-effects (LME) models and *multcomp* [38] to perform pairwise comparisons adjusted by Tukey’s test. The threshold for significance was set at *p* < 0.05. Data are presented as median (minimum, maximum) unless otherwise stated.

A first LME model included the treatments of Experiment 1 (control, SP-AI, NM) as fixed effects and the females as the random part of the model. Pairwise comparisons were adjusted by Tukey’s test. A second LME model included the different collection times of Experiment 2 (10, 24, 36, 68 and 72 h post-mating) as fixed effects and the females as the random part of the model. Post hoc comparisons were performed using Tukey’s multiple comparisons test.

The differential expression changes in qPCR results among tissues in both Experiments were re-analyzed separately. The utero-tubal junction was used as an arbitrary anatomical compartment reference among all tissues examined to compare the gene expression changes per gene [34], issued both by control, NM or SP-AI (Experiment 1) or by different times post-mating (Experiment 2). The LME model was followed by Tukey’s multiple comparison test to analyze the differences among each anatomical region of the female reproductive tract. Data are presented as median (minimum, maximum) unless otherwise stated. Data on the differential expression among tissues are presented as Row Z-scores.

## 3. Results

### 3.1. Natural Mating and Sperm-Free Seminal Plasma Infusion Differentially Modulated Anti-Inflammatory Cytokines and VEGF-A Genes

**Figure 2:**
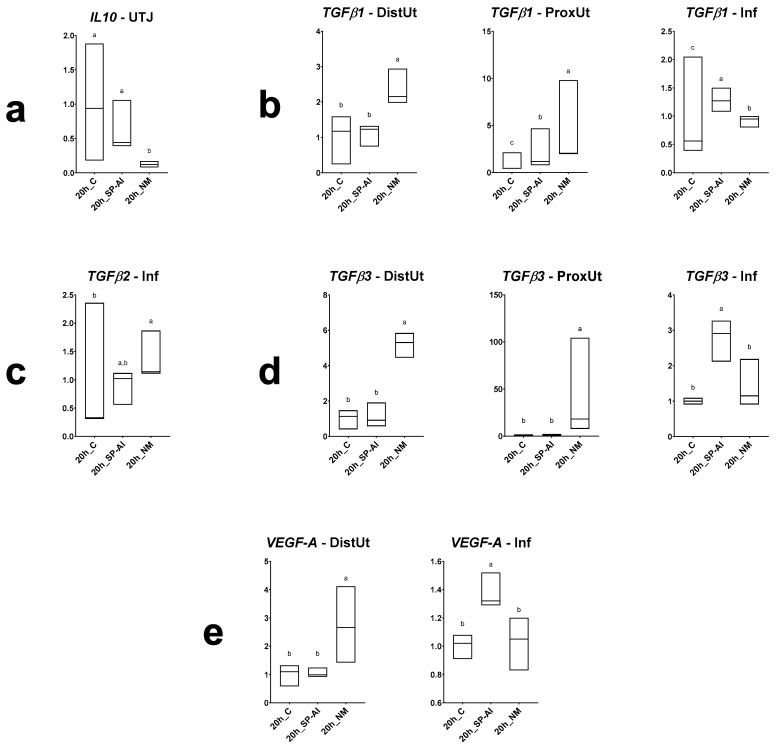
Results of Experiment 1. Gene expression of (**a**) *IL10*, (**b**) *TGFβ1*, (**c**) *TGFβ2*, (**d**) *TGFβ3* and (**e**) *VEGF-A* in differentially expressed segments (*p* < 0.05) (DistUt: distal uterus, ProxUt: proximal uterus, UTJ: utero-tubal junction, Amp: ampulla and Inf: infundibulum) of the does internal reproductive tract after 20 h of the induction of the ovulation with 0.03 mg gonadotropin-releasing hormone (GnRH) intramuscularly (20 h_C, *n* = 3), 20 h post-GnRH-stimulation and seminal plasma vaginal infusion (20h_SP-AI, *n* = 3) and 20 h post-GnRH-stimulation and natural mating (20 h_NM, *n* = 3). Fold changes relative to the control of the ovulation group are shown. Different letters (a,b) represent statistical differences between groups (*p* < 0.05). Median (minimum, maximum).

Differences in *IL10*, *TGFβ1*, *TGFβ2*, *TGFβ3* and *VEGF-A* expression among groups included in Experiment 1 are displayed in Figure 2 (and Figure S1). The NM treatment downregulated the *IL10* expression in the utero-tubal junction (*p* < 0.05); upregulated the *TGFβ1* and *TGFβ3* expression in the distal (*p* < 0.01) and proximal uterus (*p* < 0.001); upregulated the *TGFβ1* and *TGFβ2* expression in the infundibulum (*p* < 0.01); and upregulated the *VEGF-A* expression in the distal uterus (*p* < 0.05). The SP-AI treatment upregulated the *TGFβ1* expression in the proximal uterus (*p* < 0.01), upregulated the *TGFβ1* and *TGFβ3* expression in the infundibulum (*p* < 0.01), and upregulated the *VEFG-A* expression in the infundibulum (*p* < 0.01).

### 3.2. Temporal Gene Expression of Anti-Inflammatory cytokines and VEGF-A at 10 h up to 72 h Post-Mating

**Figure 3:**
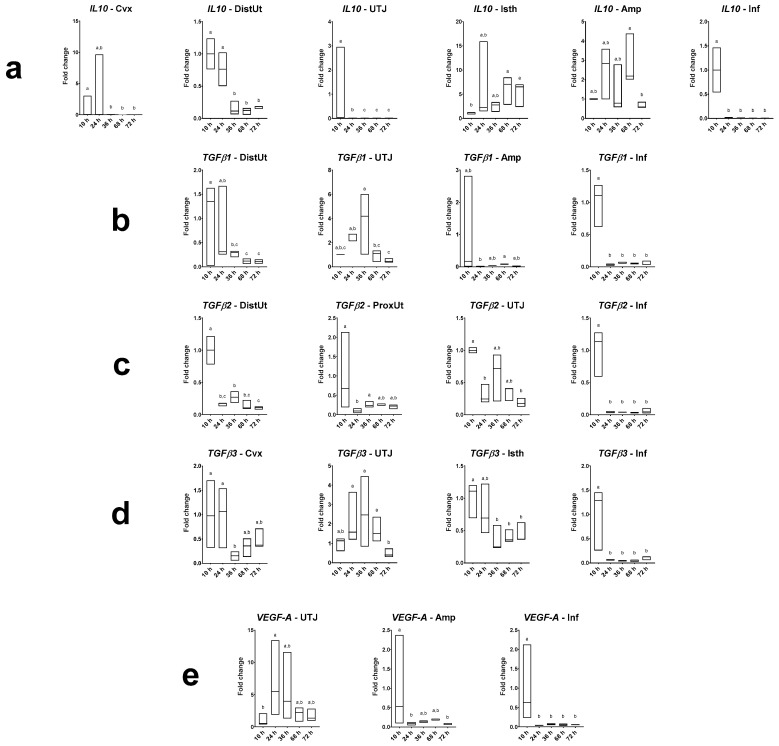
Results of Experiment 2. Gene expression of (**a**) *IL10*, (**b**) *TGFβ1*, (**c**) *TGFβ2*, (**d**) *TGFβ3* and (**e**) *VEGF-A* was statistically significant in rabbit endocervix (Cvx), distal uterus (DistUt), proximal uterus (ProxUt), utero-tubal junction (UTJ), distal isthmus (Isth), ampulla (Amp) and infundibulum (Inf) after 10, 24, 36, 68 or 72 h post-induction of the ovulation (intramuscular injection of 0.03 mg gonadotropin-releasing hormone) and natural mating (*n* = 3/collection time). Fold changes relative to 10 h post-mating group are shown. Different letters (a–c) represent statistical differences between groups (*p* < 0.05). Median (minimum, maximum).

Differences in *IL10*, *TGFβ1*, *TGFβ2*, *TGFβ3* and *VEGF-A* expression in does sampled at different times post-mating are displayed in Figure 3 (and Figure S2), with 10 h post-mating as the control group. The *IL10* expression was downregulated at 36, 68 and 72 h post-mating in the endocervix and distal uterus (*p* < 0.001); downregulated at 24, 36, 68 and 72 h post-mating in the utero-tubal junction and infundibulum (*p* < 0.001); and upregulated at 68 and 72 h post-mating in the distal isthmus (*p* < 0.05). The *TGFβ1* expression was downregulated at 36, 68 and 72 h post-mating in the distal uterus (*p* < 0.05); and downregulated at 24, 36, 68 and 72 h post-mating in the infundibulum (*p* < 0.001). The *TGFβ2* expression was downregulated at 24, 36, 68 and 72 h post-mating in the distal uterus and infundibulum (*p* < 0.001); downregulated at 24 h post-mating in the proximal uterus (*p* < 0.01); and downregulated at 24 and 72 h post-mating in the utero-tubal junction (*p* < 0.05). The *TGFβ3* expression was downregulated at 36 h post-mating in the endocervix (*p* < 0.05); downregulated at 36, 68 and 72 h post-mating in the distal isthmus (*p* < 0.05); and downregulated at 24, 36, 68 and 72 h post-mating in the infundibulum (*p* < 0.001). The *VEGF-A* expression was upregulated at 24 h post-mating in the utero-tubal junction (*p* < 0.05); downregulated at 24 and 72 h post-mating in the ampulla (*p* < 0.05); and downregulated at 24, 36, 68 and 72 h post-mating in the infundibulum (*p* < 0.001).

### 3.3. Spatial Gene Expression Triggered by Natural Mating and Sperm-Free Seminal Plasma Infusion

**Figure 4:**
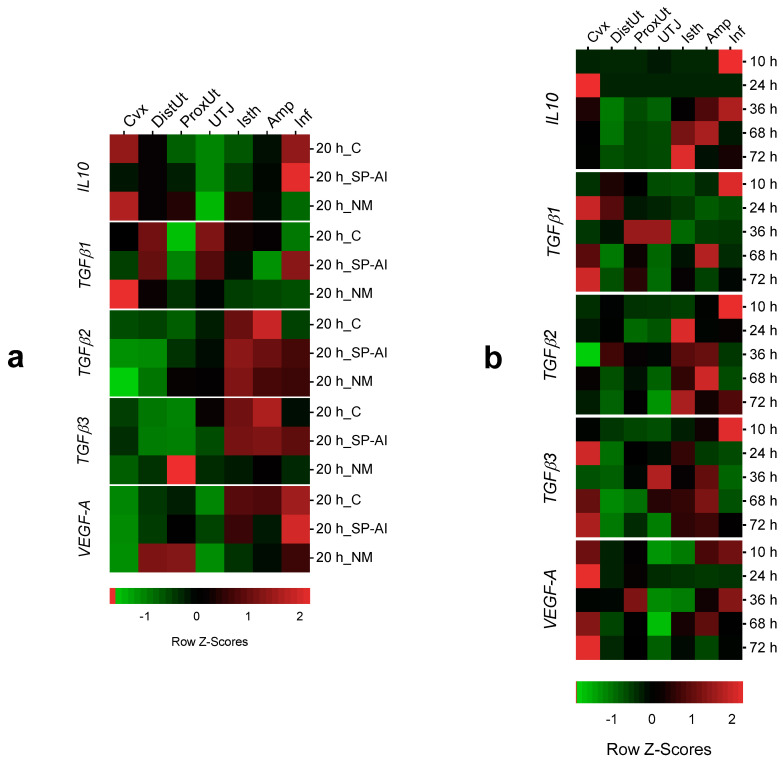
Heatmap composition of the gene expression changes among tissues in the different groups included in the study. Changes in *IL10*, *TGFβ1*, *TGFβ2*, *TFGβ3* and *VEGF-A* expression among tissues (endocervix, Cvx; distal uterus, DistUt; proximal uterus, ProxUt; utero-tubal junction, UTJ; distal isthmus, Isth; ampulla, Amp; and infundibulum, Inf) (**a**) at 20 h after the induction of ovulation with 0.03 mg of gonadotropin-releasing hormone (GnRH) intramuscularly, as the control of ovulation (20 h_C); 20 h post-GnRH stimulation and sperm-free seminal plasma vaginal infusion (20 h_SP-AI); and 20 h post-GnRH stimulation and natural mating (20 h_NM); (**b**) at 10, 24, 36, 68 and 72 h post-GnRH-stimulation and natural mating. Row Z-scores of the mean fold change relative to the reference group (UTJ) are shown. Red indicates upregulation, and green indicates downregulation.

The tissue analysis expression showed significant differences in *IL10*, *TGFβ1*–3 and *VEGF-A* expression among tissues in the different groups included in Experiment 1 (Figure 4a and Figure S3). The infundibulum presented the highest *IL10* expression in the SP-AI and control groups (*p* < 0.05), whereas the cervix presented the highest *IL10* expression in the NM group (*p* < 0.05). In the case of *TGFβ1* expression, the endocervix presented the highest expression in the NM group (*p* < 0.05). The distal isthmus presented the highest *TGFβ2* expression in all groups included in Experiment 1 (*p* < 0.05). Similarly, the oviductal tissues presented the highest *TGFβ3* expression in the SP-AI and control groups (*p* < 0.05). However, the proximal uterus presented the highest *TGFβ3* expression in the NM group (*p* < 0.01). The oviductal tissues presented the highest *VEGF-A* expression in the SP-AI and control groups (*p* < 0.05), whereas the uterine tissues presented the highest *VEGF-A* expression in the NM group (*p* < 0.05).

### 3.4. Spatial Gene Expression at 10 h up to 72 h Post-Mating

The tissue analysis expression showed significant differences in *IL10*, *TGFβ1–3* and *VEGF-A* expression among tissues in the different groups included in Experiment 2 (Figure 4b and Figure S4). At 10 h post-mating (time of ovulation), the infundibulum presented the highest *IL10* expression (*p* < 0.01). Conversely, at 24 h post-mating, the endocervix presented the highest *IL10* expression (*p* < 0.05). At 36, 68 and 72 h post-mating, the oviductal segments presented higher *IL10* expression compared to the uterine tissues (*p* < 0.05).

At 10 h post-mating, the infundibulum presented the highest *TGFβ1–3* expressions (*p* < 0.05). The *TGFβ1* expression was higher in the uterine tissues compared to the oviduct segments (*p* < 0.05), whereas the *TGFβ2* expression was higher in the oviduct segments compared to the uterine tissues (*p* < 0.05). Both uterine and oviductal segments presented the highest *TGFβ3* expressions (*p* < 0.05). The uterine tissues presented higher *VEGF-A* expressions (*p* < 0.05) compared to oviduct segments. However, at 10 and 36 h post-mating, the infundibulum presents the highest *VEGF-A* expression (*p* < 0.05).

## 4. Discussion

The results showed a differential expression triggered by sperm-free SP infusion and natural mating on anti-inflammatory cytokines and angiogenesis-related genes, as well as the differential expression produced at different times post-mating in the rabbit, an induced ovulatory species. Additionally, the study provides the differential expression on anti-inflammatory cytokines and angiogenesis-related genes along the doe reproductive tract triggered by natural mating and sperm-free SP infusion, as well as by preimplantation embryo at different times post-mating.

The SP interacts with the female reproductive tract-inducing female immune adaptation processes required to tolerate male antigens suppressing inflammation and immune rejection responses [10]. This maternal immune tolerance is essential for ongoing pregnancy success [39]. Additionally, the SP induces molecular and cellular modifications in the uterus and in the highest reproductive tract promoting embryo development and implantation competence [10]. In this regard, different soluble components present in the SP play key roles in these processes, such as the TGFβs, which can drive immune cells to tolerogenic phenotypes [40]. This study revealed differences between sperm-free SP and natural mating (that also contains SP) in the *IL10*, *TGFβ1*, *TGFB3* and *VEGF-A* expression. The data suggest that natural mating, at 20 h post-exposure, downregulated the *IL10* expression in the utero-tubal junction, probably related to the spermatozoa selection or clearance in this region. Conversely, the *TGFβ1*, *TGFβ3* and *VEGF-A* expressions were upregulated in the uterine tissues in the natural mating group, whereas upregulated in the infundibulum in the sperm-free SP infusion group.

Even though the SP appears to not reach the upper segments of the oviduct after mating, it seems that the signaling originated in lower segments travel to upper segments in response to SP [10]. The range of effects produced by SP in different species includes clearance of microorganisms in the uterus after mating, sperm selection, induction of ovulation, the formation of corpus luteum and supporting the development of the preimplantation embryo, among others [10]. Our observations indicate that the sperm-free SP infusion increased the expression of *TGFβ1*, *TGFβ3* and *VEGF-A* in the infundibulum, whereas the expression of these genes in the natural mating group, also containing SP, remained unaltered compared to the control group. Moreover, the natural mating group increased *VEGF-A* expression in the distal uterus, but not in the oviduct, suggesting that the rabbit uterus reacts to post-mating inflammation [15], likely to process seminal material and recovery for uterine tissue homeostasis after mating. Our data suggest that the responsiveness of the doe reproductive tract differs between the sperm-free SP and sperm-containing SP treatments. The signaling originated by the sperm-free SP infusion was able to travel to upper segments of the oviduct, as previously demonstrated in several species [10], whereas the signal generated by the natural mating group remained in the uterine tissues. This pattern was also observed in the temporal tracking experiment of the study, suggesting that the rabbit uterus may react to the early stages of embryo development before its arrival from the oviduct, but the mechanism involved is yet not fully understood.

The TGFβs isoforms present a diverse range of functions in reproductive tissues, including gonad development, gamete production, embryo implantation, fetal and placental development, development of secondary sex organs and immune tolerance of gametes and conceptus antigens [22]. The effects of TGFβs are mediated by antigen-presenting cells such as macrophages and dendritic cells, creating and maintaining an immunotolerant environment for the conceptus antigens if an anti-inflammatory dominance is established [22]. In this regard, our results revealed an upregulation of *TGFβs* expression at 20 h post-mating in the endometrium, suggesting that mating induces suppression of anti-sperm immune response [39]. Similar results were found at 10 h post-mating in oviductal segments, probably related to the ovulation that takes place 10 h after coitus or GnRH stimulation [7] or the suppression of anti-sperm immune response induced by the transport of spermatozoa through the oviduct before fertilization [41,42]. However, the upregulation of *TGFβs* expression in the oviduct was not present in the rest of the times after mating compared to 10 h post-mating, when the early embryo stages of development are present along the oviduct [43]. Thus, the effect produced by semen on the *TGFβs* expression changes seemed to be stronger than the effect of the embryo transport through the oviduct at different stages of development, presumably due to the different number of cells in contact with the female reproductive tract (300 million spermatozoa [44] vs. 4–12 embryos [45]). Although the effects of spermatozoa and the early embryo stages of development on *TGFβs* expression seems to differ in magnitude, tissue analyses revealed a *TGFβ2* and *TGFβ3* expression pattern in the oviduct, increasing the expression of those genes. Moreover, the *TGFβ1* expression pattern in the uterus in the presence of spermatozoa and the early stages of embryo development may indicate that both antigenic compounds induced an anti-inflammatory response creating and maintaining an immunotolerance environment in the oviduct and uterus, essential for the pregnancy success [39]. Similarly, this immunotolerant environment to spermatozoa may be observed by the upregulation of the *IL10* expression in the endocervix and distal uterus at 10 and 24 h post-mating.

The tissue and temporal analyses revealed an interesting pattern along the oviduct during the preimplantation embryo transport. When fertilization occurs in the ampulla [46], embryos are delayed at the ampulla–isthmus junction until 48 h post-mating and then pass through the isthmus portion after 70 h post-mating to finally reach the utero-tubal junction and enter the uterus [43]. Our observations showed an upregulation of *TGFβs* expression in the infundibulum at the time of induced ovulation (10 h post-mating), similarly, as the pattern observed in the *IL10* expression at the same time, might relate with the inflammation process associated with ovulation [47]. At 24 h post-mating, the endocervix displayed the highest expression of *IL10, TGFβ1, TGFβ3* and *VEGF-A*, whereas the *TGFβ2* expression was higher in the isthmus compared to the endocervix. Although fertilization takes place at 12–13 h post-mating [46], this upregulation of anti-inflammatory cytokines and angiogenesis mediators in the endocervix may be related to preserving spermatozoa viability and fertilization competence [13], in accordance with previous research that proposed the cervix as the first anatomical barrier for spermatozoa reservoir in rabbits [20,48,49].

The coordination between the preimplantation embryos and the differentiation of the uterus to a receptive state must be closely synchronized for successful embryo implantation [50]. Following the stromal and epithelial cell changes, the endometrial vascular bed undergoes a marked expansion before implantation [50,51]. This vascular expansion, in size and number, is markedly enhanced during implantation and placental structure development [51]. In the rabbit, the vascular supply during pregnancy is produced by the addition of new vessels and the expansion of the existing ones [52]. Our results revealed that *VEGF-A* expression was upregulated at 10 h post-mating in the infundibulum and ampulla, probably related to the ongoing processes like ovulation [7] and the imminent fertilization [46]. The *VEGF-A* expression displayed higher relative expression values at 20 h post-mating in the endometrium compared to the oviduct, perhaps related to preparing the receptive endometrium for the embryo implantation. However, this effect was not observable at different times post-mating, suggesting that additional factors, other than the analyzed in our experimental setup, are involved in the differentiation of the uterus to a receptive state before embryos reach the uterus.

## 5. Conclusions

Our results confirmed a differential modulation of the expression of anti-inflammatory and angiogenesis-related-genes by sperm-free SP and natural mating. Moreover, the gene expression analysis up to 72 h after induction of the ovulation might suggest a concerted effect of the presence of sperm and preimplantation embryos in the differential expression of these genes along the reproductive tract of the doe. Our findings warrant further research both to discriminate the effects of mating, spermatozoa, SP and preimplantation embryos on the reproductive tract of the doe and to fully describe the mechanism involved in such differential gene expression. Thus, mimicking the gene modulations induced by natural mating and sperm-free SP using physiological strategies in the current rabbit breeding systems may enhance the efficiency, productivity and sustainability of such systems.

## Figures and Tables

**Table 1:** Primers used for the quantitative PCR analyses.

Gene	Primer Sequence (5′–3′)	Product Size (bp)	Efficiency (%)
*β-ACTIN*	F: commercial, not available	120	88.6
	R: commercial, not available		
*IL10*	F: TTGTTAACCGAGTCCCTGCT	209	107.5
	R: TTTTCACAGGGGAGAAATCG		
*TGFβ1*	F: CACCATTCACAGCATGAACC	129	99.3
	R: TTCTCTGTGGAGCTGAAGCA		
*TGFβ2*	F: CCGGAGGTGATCTCCATCTA	236	94.7
	R: AGGTACCCACAGAGCACCTG		
*TGFβ3*	F: CACGAACCCAAGGGCTACTA	186	97.5
	R: GGTCCTCCCGACGTAGTACA		
*VEGF-A*	F: GGAGACAATAAACCCCACGA	219	87.0
	R: CTGCATGGTGACGTTGAACT		

*β-ACTIN*: β-actin, *IL10*: interleukin 10, *TGFβ1*: transforming growth factor β 1, *TGFβ2:* transforming growth factor β 2, *TGFβ3*: transforming growth factor β 3, *VEGF-A*: vascular endothelial growth factor A, F: forward, R: reverse, A: adenine, C: cytosine, G: guanine, T: thymine, bp: base pair.

## Supplementary Materials

The following are available online at https://www.mdpi.com/2076-2615/10/12/2207/s1, Figure S1: Changes in (a) *IL10*, (b) *TGFβ1*, (c) *TGFβ2*, (d) *TGFβ1* and (e) *VEGF-A* expression among different treatments at 20 h post-treatment: 20 h post-induction of the ovulation, control (20 h_C); 20 h post-seminal plasma infusion, 20 h_SP; and 20 h post-natural mating, 20 h_NM. Tissue anatomical regions of the rabbit female reproductive tract (endocervix, Cvx; distal uterus, DistUt; proximal uterus, ProxUt; utero-tubal junction, UTJ; distal isthmus, Isth; ampulla, Amp; and infundibulum, Inf).-fold changes relative to the reference group (20 h_C) are shown. Different letters (a–c) represent statistical differences between tissues (*p* < 0.05). Median (minimum, maximum); Figure S2: Changes in (a) *IL10*, (b) *TGFβ1*, (c) *TGFβ2*, (d) *TGFβ1* and (e) *VEGF-A* expression at different times (from 10 to 72 h) post-mating: 10, 24, 36, 68 and 72 h post-natural mating. Tissue anatomical regions of the female rabbit reproductive tract (endocervix, Cvx; distal uterus, DistUt; proximal uterus, ProxUt; utero-tubal junction, UTJ; distal isthmus, Isth; ampulla, Amp; and infundibulum, Inf).-fold changes relative to the reference group (10 h post-mating) are shown. Different letters (a–c) represent statistical differences between tissues (*p* < 0.05). Median [minimum, maximum]; Figure S3: Changes in (a) *IL10*, (b) *TGFβ1*, (c) *TGFβ2*, (d) *TGFβ1* and (e) *VEGF-A* expression among different tissues at 20 h post-treatment. 20 h post-induction of the ovulation, control (20 h_C); 20 h post-seminal plasma infusion, 20 h_SP; and 20 h post-natural mating, 20 h_NM. Tissue anatomical regions of the female rabbit reproductive tract (endocervix, Cvx; distal uterus, DistUt; proximal uterus, ProxUt; utero-tubal junction, UTJ; distal isthmus, Isth; ampulla, Amp; and infundibulum, Inf).-fold changes relative to the reference group (UTJ) are shown. Different letters (a–d) represent statistical differences between tissues (*p* < 0.05). Median [minimum, maximum]; Figure S4: Changes in (a) *IL10*, (b) *TGFβ1*, (c) *TGFβ2*, (d) *TGFβ1* and (e) *VEGF-A* expression among different tissues in the period 10–72 h (10, 24, 36, 68 and 72 h post-natural mating). Tissue anatomical regions of the female rabbit reproductive tract (endocervix, Cvx; distal uterus, DistUt; proximal uterus, ProxUt; utero-tubal junction, UTJ; distal isthmus, Isth; ampulla, Amp; and infundibulum, Inf).-fold changes relative to the reference group (UTJ) are shown. Different letters (a–d) represent statistical differences between tissues (*p* < 0.05). Median [minimum, maximum].
