# Supplementary material for: Semen Modulates Inflammation and Angiogenesis in the Reproductive Tract of Female Rabbits
Source: Animals (Basel). 2020 Nov 25;10(12):2207. doi: 10.3390/ani10122207 (PMC7761520; doi:10.3390/ani10122207)
Supplement: Supplementary file 1 [file animals-10-02207-s001.pdf]

animals

MDPI

Article

Semen Modulates Inflammation and Angiogenesis in the Reproductive Tract of Female Rabbits

Jaume Gardela [1,2], Amaia Jauregi-Miguel [3], Cristina A. Martinez [1], Heriberto Rodríguez-Martinez [1], Manel López-Bejar [2,4] and Manuel Álvarez-Rodríguez [1,2,*]

1 Department of Biomedical and Clinical Sciences (BKV), Division of Children's and Women Health (BKH), Obstetrics and Gynecology, Linköping University, 58185 Linköping, Sweden; jaume.gardela@uab.cat (J.G.); cristina.martinez-serrano@liu.se (C.A.M.); heriberto.rodriguez-martinez@liu.se (H.R.-M.)

2 Department of Animal Health and Anatomy, Veterinary Faculty, Universitat Autònoma de Barcelona, 08193 Bellaterra, Spain; manel.lopez.bejar@uab.cat

3 Division of Molecular Medicine and Virology (MMV), Linköping University, 58185 Linköping, Sweden; amaya.jauregi.miguel@liu.se

4 College of Veterinary Medicine, Western University of Health Sciences, Pomona, CA 91766, USA

* Correspondence: manuel.alvarez-rodriguez@liu.se

Received: 19 October 2020; Accepted: 18 November 2020; Published: date

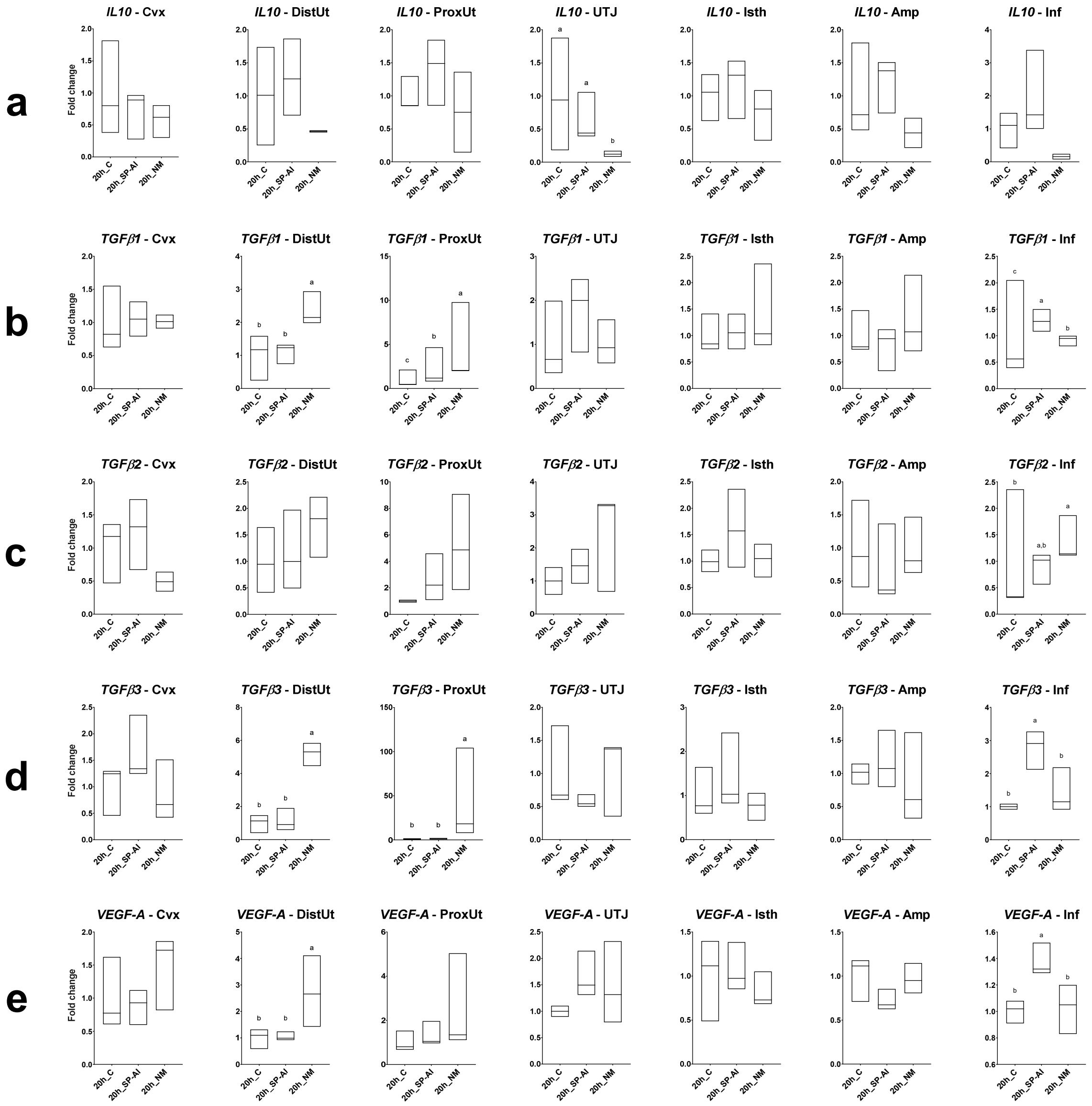

Figure S1. Changes in **a**) *IL10*, **b**) *TGFβ1*, **c**) *TGFβ2*, **d**) *TGFβ1*, and **e**) *VEGF-A* expression among different treatments at 20 h post-treatment: 20 h post-induction of the ovulation, control (20 h_C); 20 h post-seminal plasma infusion, 20 h_SP; and 20 h post-natural mating, 20 h_NM. Tissue anatomical regions of the rabbit female reproductive tract (endocervix, Cvx; distal uterus, DistUt; proximal uterus, ProxUt; utero-tubal junction, UTJ; distal isthmus, Isth; ampulla, Amp; and infundibulum, Inf). Fold changes relative to reference group (20 h_C) are shown. Different letters (a-c) represent statistical differences between tissues ($p < 0.05$). Median (minimum, maximum).

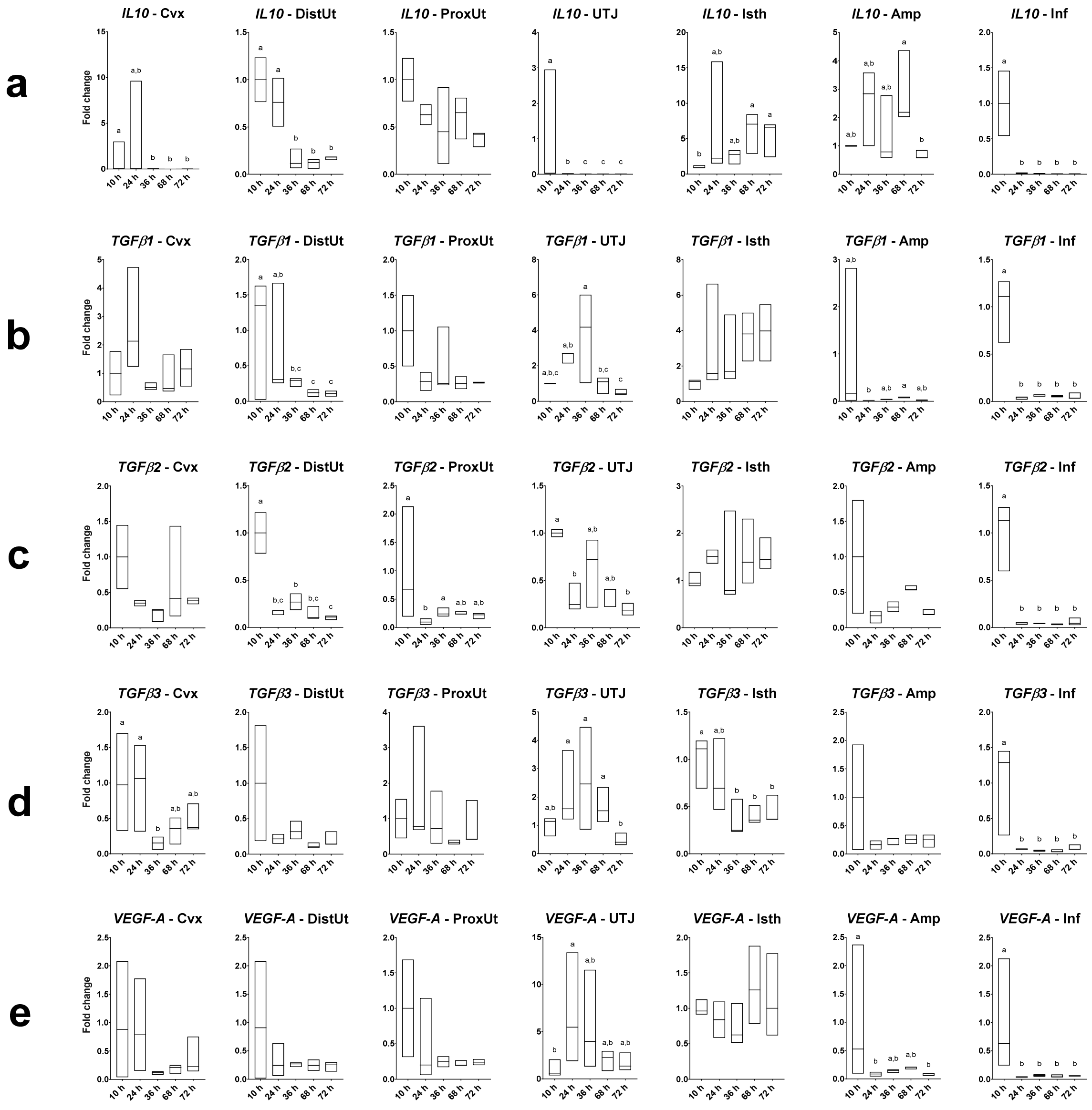

Figure S2. Changes in **a**) *IL10*, **b**) *TGFβ1*, **c**) *TGFβ2*, **d**) *TGFβ1*, and **e**) *VEGF-A* expression at different times (from 10 to 72 h) post-mating: 10, 24, 36, 68, and 72 h post-natural mating. Tissue anatomical regions of the female rabbit reproductive tract (endocervix, Cvx; distal uterus, DistUt; proximal uterus, ProxUt; utero-tubal junction, UTJ; distal isthmus, Isth; ampulla, Amp; and infundibulum, Inf). Fold changes relative to the reference group (10 h post-mating) are shown. Different letters (a–c) represent statistical differences between tissues ($p < 0.05$). Median (minimum, maximum).

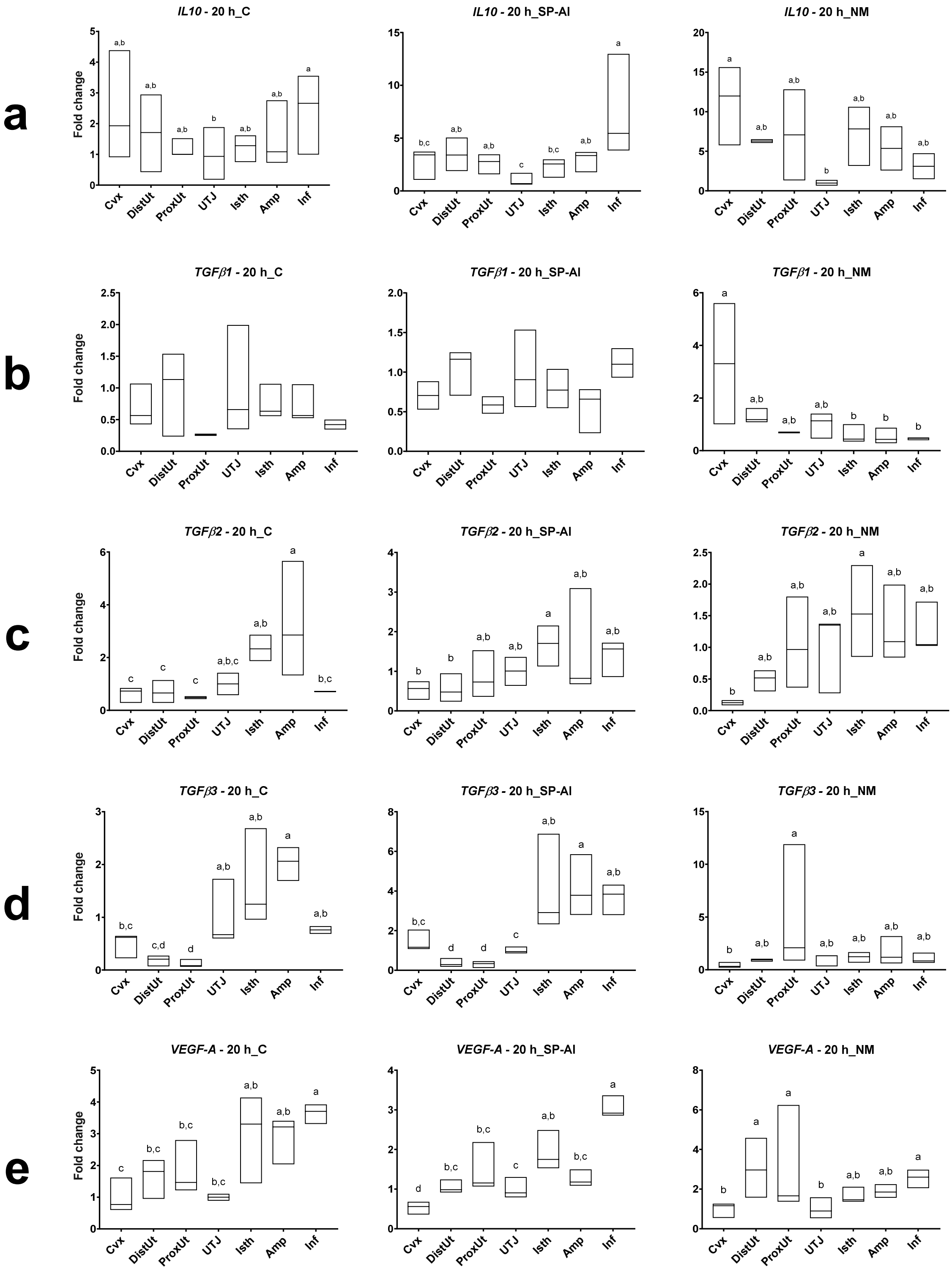

Figure S3. Changes in **a**) *IL10*, **b**) *TGFβ1*, **c**) *TGFβ2*, **d**) *TGFβ1*, and **e**) *VEGF-A* expression among different tissues at 20 h post-treatment. 20 h post-induction of the ovulation, control (20 h_C); 20 h post-seminal plasma infusion, 20 h_SP; and 20 h post-natural mating, 20 h_NM. Tissue anatomical regions of the female rabbit reproductive tract (endocervix, Cvx; distal uterus, DistUt; proximal uterus, ProxUt; utero-tubal junction, UTJ; distal isthmus, Isth; ampulla, Amp; and infundibulum, Inf). Fold changes relative to the reference group (UTJ) are shown. Different letters (a–d) represent statistical differences between tissues ($p < 0.05$). Median (minimum, maximum).

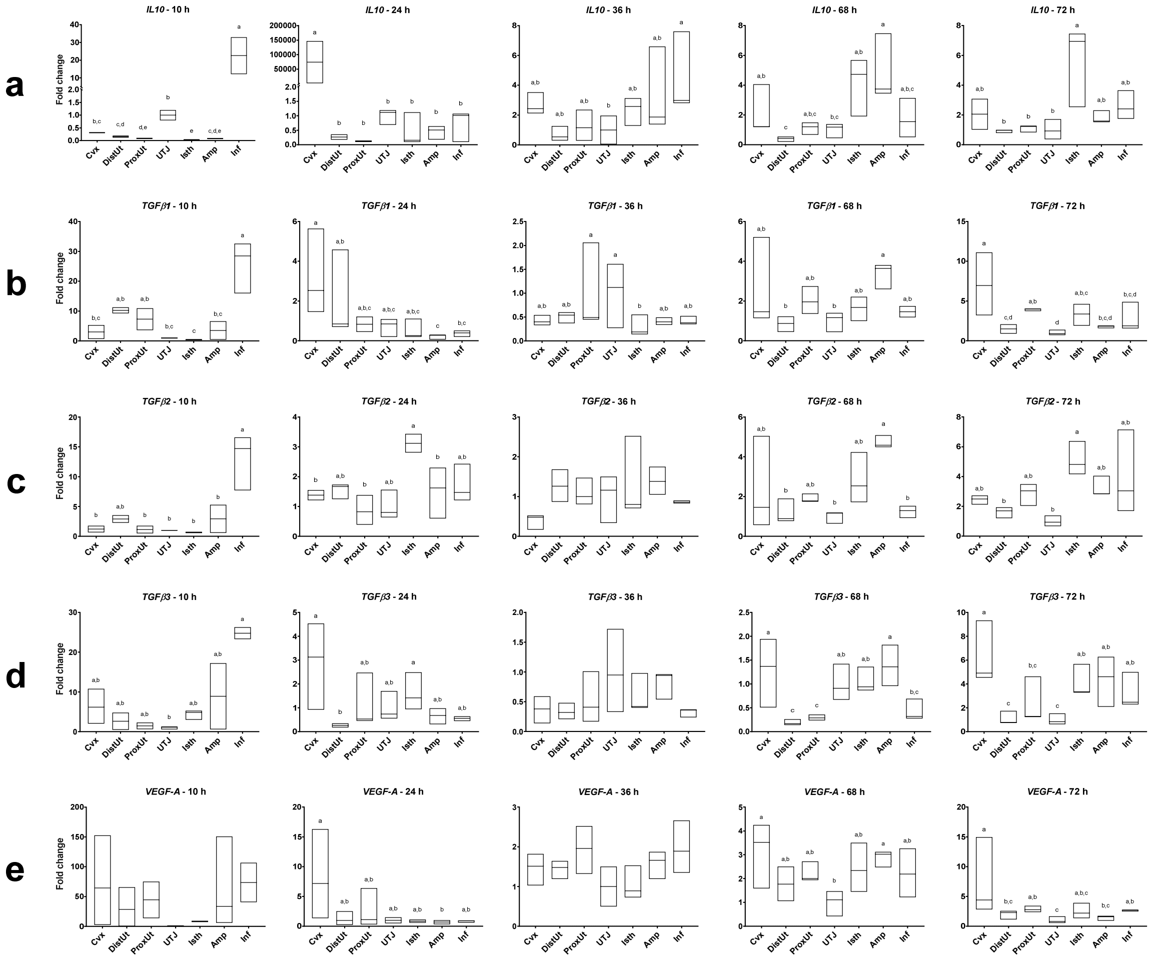

Figure S4. Changes in **a**) *IL10*, **b**) *TGFβ1*, **c**) *TGFβ2*, **d**) *TGFβ1*, and **e**) *VEGF-A* expression among different tissues in the period 10–72 h (10, 24, 36, 68, and 72 h post-natural mating). Tissue anatomical regions of the female rabbit reproductive tract (endocervix, Cvx; distal uterus, DistUt; proximal uterus, ProxUt; utero-tubal junction, UTJ; distal isthmus, Isth; ampulla, Amp; and infundibulum, Inf). Fold changes relative to the reference group (UTJ) are shown. Different letters (a–d) represent statistical differences between tissues ($p < 0.05$). Median (minimum, maximum).
